# Polio Eradication in Pakistan: Innovation and Digitalization—the Key to Crossing the Finish Line

**DOI:** 10.3389/phrs.2024.1608084

**Published:** 2025-01-03

**Authors:** Amna Awan

**Affiliations:** Primary and Secondary Healthcare Department, Punjab, Lahore, Pakistan

**Keywords:** polio eradication, microplanning, innovation framework, GIS mapping, GPS tracking of vaccinators

## Introduction

The global polio eradication initiative (GPEI), which started as a resolve from World Health Organization (WHO) leaders in 1988 to eradicate polio globally, has been labelled as the largest international health initiative to date. Billions of dollars and thousands of employees have been deployed worldwide since its conception, with the singular aim of eradicating this disease [1]. The first 14 years of this program saw huge success as indigenous polio was eradicated in all but six countries in the world [2].

The current situation in 2024 is that polio has been eliminated in all but two countries of the world, the exceptions being Pakistan and Afghanistan. Nigeria was the latest country to achieve elimination status in 2020 [3].

This paper discusses the introduction of **innovative technology** to address the current gaps in Pakistan’s polio program. **Public Health Innovation Framework** has been proposed in the paper as a guiding tool for the introduction of this innovation to the polio program of Pakistan.

## Problem Statement

Pakistan has failed to achieve polio-free status despite monumental international donor support, advocacy for the program, and long-standing commitment from the country to the program. By failing to achieve polio-free status in the last decade, the country has missed an important window. This has made the achievement of future targets more difficult due to new challenges such as population explosion, climate change, economic recession and vaccine hesitancy in the post-COVID era, misinformation, social media, and increased inequality [4].

These newer challenges mean that new solutions must be introduced to tackle old problems. However, the Pakistan polio program has not been updated in many years. The supplementary immunization activities (SIAs) and polio campaigns are paper-based and outdated, and no attempt has been made to digitalize the program [4].

The PEI program in Pakistan was on track, with significant progress being made from 2015. However, the **surge of cases in 2019 followed by the COVID-19 pandemic** proved to be enough to derail progress. The halting of activities and transfer of polio resources to COVID-19 vaccinations resulted in an increased number of cases in 2020. The number of cases decreased in 2021, either due to relaxed surveillance or restriction of inter- and cross-border movements due to lockdowns. The current situation in Pakistan is that 40 confirmed WPV cases have been notified since 24 Oct 2024, and **environmental samples** are continuously positive in many urban and peri-urban areas of Pakistan [5]. This signifies continuous transmission of the virus in the communities, due to the presence of unvaccinated **“missed children**.**”** In order to achieve elimination status, the country’s focus should be on reaching and vaccinating these missed children, who are the hidden foci of transmission. This can be achieved by **improving the quality rather than the quantity of our SIAs**, which is contrary to the current norm [6].

As mentioned earlier, we are in unprecedented times and are facing problems with no current known solutions; therefore, this is the **best time to experiment with digital technology**.

## Framework for Public Health Innovation

The tool used to introduce our proposed innovation is the Framework for Public Health Innovation [7]. The innovation being proposed is the introduction of **GIS mapping in the micro-planning phase and using GPS technology to track paths of vaccinators in SIAs and National Immunization Days. Nigeria**, the last country to eliminate polio in 2020, has introduced this GPS/GIS technology in their NIDs [8].

## Status Quo

As Pakistan has failed to achieve their elimination target, and with the impending threat of funding withdrawal, it is evident that the current elimination efforts in Pakistan are not enough and need to be supplemented by innovative methods. Thus, this climate provides an ideal playground to introduce evidence-based innovation.

## Type of Innovation

### Mapping of Areas for Outreach Activity Using the Geographic Information System

GIS mapping has been identified as an effective strategy to identify remote and hard-to-reach areas. UNICEF and GAVI recommend GIS mapping to identify zero-dose and missed children. GIS technology would also assist in identifying chronically missed settlements and would help in concentrating efforts to these high-risk areas during the SIAs [9].

**FIGURE 1:**
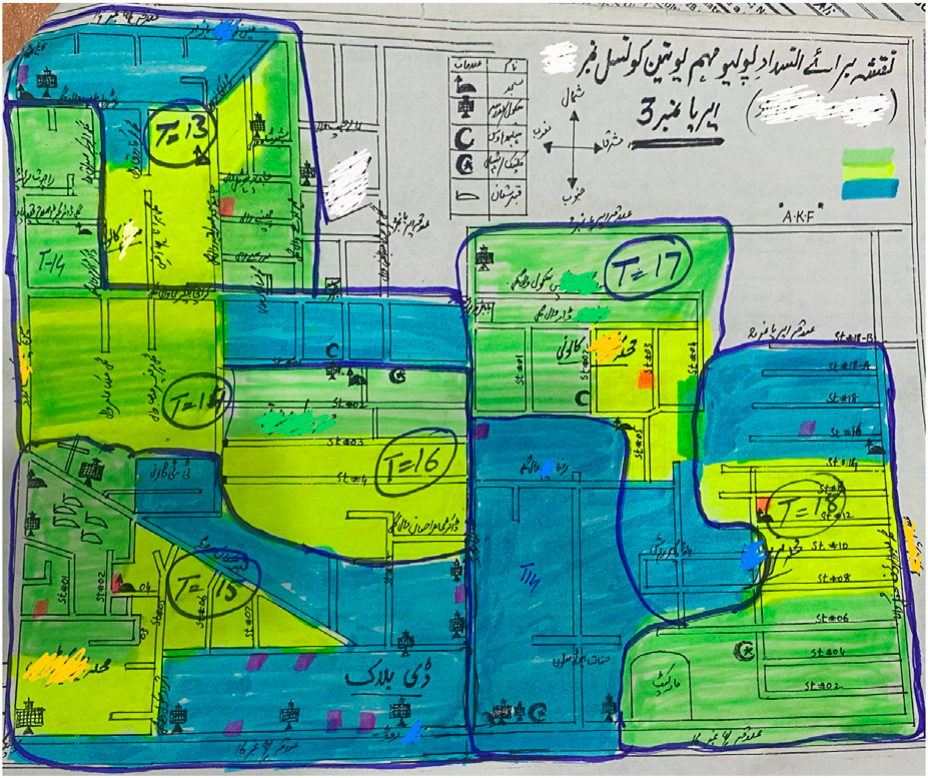
Sample Hand drawn map used during micro-planning in Pakistan’s Polio Program.

As shown in Figure 1, maps are **hand-drawn by polio teams** during the microplanning phases at union council level. These maps are of **low quality** and do not provide information like geographical complexity or hard-to-reach areas. Thus, these maps have low utility in practice and serve no purpose other than filling a formal requirement by WHO. On the contrary, GIS maps used for immunization in Chad gave the exact location of settlements, and planners have identified the location of missed populations using this map [10].

### GPS Tracking of Vaccinators in the Field

A GPS system would aid in monitoring the campaigns. A web-based tracking dashboard was developed in Nigeria that tracked the vaccinators through a GPS tracking system during campaigns; this could be simulated in Pakistan’s polio SIAs. Such a tracking system would aid in the supervision of vaccinators as well as in identification of missed pockets [8].

The GIS/GPS tracking process can be introduced in a sequential manner.

## First Stage

The innovation should be piloted in high-risk districts of **Sindh and Baluchistan**, where most polio cases have been reported.

## Second Stage

A nationwide SIA should be planned, targeting the **high-risk mobile population (HRMP)**. This would include the nomadic population who do not come under formal health systems. A **targeted campaign** would be launched all over the country using the results and gains achieved in the first stage.

## Third Stage

Digitalization would be formally incorporated into the polio program.

## Partnerships

WHO and UNICEF, being the main partners, would work in collaboration with the districts. However, the main ownership of the initiative would belong to governmental bodies. Ownership of the innovation is essential to achieve sustainable gains, as seen in polio elimination in India and Nigeria.

## Accountability

The introduction of technology would involve the use of resources; thus, strict accountability for governmental agencies should be introduced to the program. **Independent third parties with no conflict of interest** should be involved in the accountability process [8].

### Conclusion

The presence of missed and zero-dose children is a major barrier to polio eradication. Weak routine immunization in the country is a gap that can be filled through operational innovation in NIDs.

There is a good system of polio teams with predefined roles at union council levels in the country. The teams have experience in using apps for data entry from previous pilot projects and in their routine jobs as well. Thus, the introduction of the innovation recommended in this report seems feasible in the current circumstances. Digitalization is the last step Pakistan’s polio initiative needs to take in order to cross the finish line.
